# Submaximal Exercise Testing in Cardiovascular Rehabilitation Settings (BEST Study)

**DOI:** 10.3389/fphys.2019.01517

**Published:** 2020-01-08

**Authors:** Jennifer L. Reed, Lisa M. Cotie, Christie A. Cole, Jennifer Harris, Bruce Moran, Kyle Scott, Tasuku Terada, John P. Buckley, Andrew L. Pipe

**Affiliations:** ^1^Division of Cardiac Prevention and Rehabilitation, University of Ottawa Heart Institute, Ottawa, ON, Canada; ^2^Faculty of Medicine, University of Ottawa, Ottawa, ON, Canada; ^3^School of Human Kinetics, Faculty of Health Sciences, University of Ottawa, Ottawa, ON, Canada; ^4^Montfort Hospital, Ottawa, ON, Canada; ^5^Centre for Active Living, University Centre Shrewsbury, Chester, United Kingdom

**Keywords:** exercise test, cardiovascular diseases, rehabilitation, physiology, submaximal

## Abstract

**Background:**

This study compared changes in measured versus predicted peak aerobic power (V̇O_2__peak_) following cardiovascular rehabilitation (CR). Peak cardiopulmonary exercise testing (CPET) results were compared to four V̇O_2__peak_ estimation methods: the submaximal modified Bruce treadmill, Astrand-Ryhming cycle ergometer, and Chester step tests, and the Duke Activity Status Index (DASI).

**Methods:**

Adults with cardiovascular disease (CVD) who completed a 12-week CR program were assessed at baseline and 12 weeks follow-up. CPET, the DASI and three subsequent submaximal exercise tests were performed in a random order.

**Results:**

Of the 50 adults (age: 57 ± 11 years) who participated, 46 completed the 12-week CR program and exercise tests. At baseline 69, 68, and 38% of the treadmill, step and cycle tests were successfully completed, respectively. At follow-up 67, 80, and 46% of the treadmill, step and cycle tests were successfully completed, respectively. No severe adverse events occurred. Significant improvements in V̇O_2__peak_ were observed with CPET (3.6 ± 5.5 mL^.^kg^–1.^min^–1^, *p* < 0.001) and the DASI (2.3 ± 4.2 mL^.^kg^–1.^min^–1^, *p* < 0.001). Bland-Altman plots of the change in V̇O_2__peak_ between CPET and the four V̇O_2__peak_ estimation methods revealed the following: a proportional bias and heteroscedastic 95% limits of agreement (95% LoA) for the treadmill test, and for the cycle and step tests and DASI, mean bias’ and 95% LoA of 1.0 mL^.^kg^–1.^min^–1^ (21.3, −19.3), 1.4 mL^.^kg^–1.^min^–1^ (15.0, −12.3) and 1.0 mL^.^kg^–1.^min^–1^ (13.8, −11.8), respectively.

**Conclusion:**

Given the greater number of successful tests, no serious adverse events and acceptable mean bias, the step test appears to be a valid and safe method for assessing group-level mean changes in V̇O_2__peak_ among patients in CR. The DASI also appears to be a valid and practical questionnaire. Wide limits of agreement, however, limit their use to predict individual-level changes.

## Introduction

Cardiovascular rehabilitation (CR) programs have been consistently shown to improve patients’ peak aerobic power (V̇O_2__peak_), which independently predicts lower all-cause and cardiovascular disease-specific mortality (Keteyian et al., 2008; Kodama et al., 2009). A one metabolic equivalent increase in V̇O_2__peak_ (1-MET = V̇O_2_ of 3.5 mL^.^kg^–1.^min^–1^) is associated with an approximate 17% and 15% decrease in all-cause and cardiovascular disease-specific mortality in men and women with coronary heart disease, respectively (Keteyian et al., 2008). The assessment of V̇O_2__peak_, as measured by symptom-limited cardiopulmonary exercise testing with ergospirometry (CPET), remains the “gold-standard” for assessing patients’ cardiopulmonary responses to CR (Canadian Association of Cardiac Rehabilitation, 2009); it is also used for risk stratification and the development of safe and effective exercise programs (Canadian Association of Cardiac Rehabilitation, 2009). CPET is, however, often impractical or impossible due to the costs, time, expertise and technological resources required (Grace et al., 2016). There remains a need to assess peak aerobic power using simple, straightforward and easily-performed evaluations in all settings. Consequently, a number of submaximal exercise tests [terminated at intensities at or below 85% of peak heart rate (HR)] and questionnaires have been developed to reduce testing costs, time, resources, and risks (Hlatky et al., 1989; Noonan and Dean, 2000; Arena et al., 2007).

Systematic reviews (Evans et al., 2015; Bennett et al., 2016) and recent original studies (Hughes and Chaturvedi, 2017; Kokkinos et al., 2017, 2018; Guo et al., 2018) have shown that submaximal exercise tests (e.g., treadmill, cycle, step, and squat tests) to predict V̇O_2__peak_ in apparently healthy adults are moderately to highly accurate. Submaximal exercise testing is widely used in the United Kingdom (Cowie et al., 2019) and increasingly promoted in low-resource settings (Grace et al., 2016). These tests are not routinely performed within most CR programs in North America, yet may provide an alternative valid, safe and highly practical approach to assessing changes in V̇O_2__peak_.

Several investigators have examined the validity of submaximal exercise tests (e.g., modified Bruce treadmill test, “warm-up” cycling, and body motion during 45-s of squatting) in predicting V̇O_2__peak_ when compared to CPET at a single time point in patients with coronary heart disease (Milani et al., 1996, 1998; Hautala et al., 2013; Papini et al., 2017), and the safety and practicality of exercise tolerance testing in CR (Simms et al., 2007). Others have also explored the validity of questionnaires in predicting V̇O_2__peak_ when compared to CPET in patients with heart disease (Rankin et al., 1996; Shaw et al., 2006). Yet, no studies have assessed changes in V̇O_2__peak_ following CR as predicted by submaximal exercise tests and a self-reported activity status questionnaire when compared to CPET in patients with cardiovascular disease (CVD).

The purpose of this study was to assess changes in V̇O_2__peak_ following CR as predicted by three submaximal exercise tests (treadmill, cycle and step tests) and a self-reported activity status questionnaire, which were validated by comparison with CPET in patients with CVD. A secondary purpose was to examine the safety of these submaximal exercise tests. We hypothesized that submaximal exercise tests would provide a valid and safe estimate of change in V̇O_2__peak_ following CR when compared to CPET in patients with CVD.

## Materials and Methods

### Study Design

**FIGURE 1:**
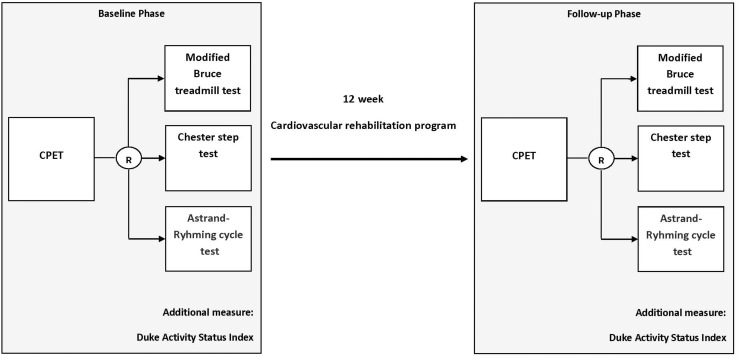
Schematic of study flow. CPET, cardiopulmonary exercise testing; R, random order.

This was a single-center pre-post experimental study (see Figure 1) conducted at the University of Ottawa Heart Institute (UOHI), a tertiary-care cardiovascular health center. This study has been described in accordance with the Strengthening the Reporting of Observational Studies in Epidemiology (STROBE) guidelines (von Elm et al., 2007). The study received ethics approval from the Ottawa Health Sciences Network Research Ethics Board (Protocol #: 20150443-01H). This study was conducted in line with the Declaration of Helsinki.

### Participants

Patients eligible for our CR program included those who have heart failure or experienced a myocardial infarction, heart surgery (e.g., coronary artery bypass graft, valve replacement or repair), heart transplantation, angioplasty and or pacemaker or defibrillator implant. Physiotherapists and nurses involved with our on-site CR program who were blinded to the study purposes referred patients who: (1) were enrolled in moderate to high functional capacity exercise classes [i.e., those who planned to exercise at ≥5 metabolic equivalents (METs)], and (2) consented to being contacted for research studies. Eligible participants were: (1) enrolled in an on-site CR program at the UOHI; (2) able to complete a symptom-limited CPET; and, (3) able and willing to provide written informed consent. Potential participants were excluded if they: (1) were unable to read and understand English or French; (2) were scheduled for a 6-min walk test (6MWT) per the established UOHI CR program algorithm (i.e., patients with very low exercise tolerance receive 6MWTs to assess their functional capacity pre-post CR); (3) were unable to complete submaximal exercise testing (i.e., treadmill, bicycle, and step) due to musculoskeletal limitations; or, (4) were currently pregnant or planning to become pregnant during the study period. All participants were recruited between November 2015 and December 2016, and provided written informed consent prior to participation.

### Exercise Testing

Participants were asked to adhere to the following instructions prior to their peak and submaximal exercise tests: (1) to take their usual medications, especially anti-arrhythmics (e.g., β-blockers) at least 1 h before testing; (2) avoid caffeine consumption at least 2 h before testing; (3) to refrain, if possible, from cigarette smoking at least 1 h before testing; (4) to void their bladder at least 30 min before testing; (5) if a diabetic patient, to have eaten or ensure adequate blood glucose levels (as per UOHI CR program policies) prior to testing; and, (6) to reschedule tests if participants felt unwell due to respiratory, gastrointestinal or febrile illness within 48 h of their appointments.

### Measured Peak Aerobic Power

V̇O_2__peak_ was measured using a symptom-limited CPET with ergospirometry on a treadmill using a ramp protocol at the beginning and conclusion of a 12-week CR program by cardiac stress technologists in the Department of Cardiac Imaging at the UOHI. This ramp protocol involves walking or jogging at a constant speed (e.g., 2.0, 3.0, or 4.0 mph) dependant on participants’ functional abilities with a 1.7% increase in grade every minute until volitional fatigue is achieved (Myers et al., 1992; American College of Sports Medicine, 2017). Gas exchange (V̇O_2_ and V̇CO_2_) was monitored continuously using a metabolic cart (Sensormedics Vmax, Yorba Linda, CA, United States), and HR was measured using ECG. The highest 10-s interval averages of V̇O_2_ and HR were considered the peak V̇O_2_ and HR values. Peak HR values were used for submaximal treadmill and step exercise test termination criteria (i.e., 85% peak HR).

### Estimated Peak Aerobic Power; Submaximal Exercise Tests

Three submaximal exercise tests (Modified Bruce treadmill, Astrand-Rhyming cycle ergometer and Chester step) were performed in a random order [using the “RAND” function of a software spreadsheet program (Excel, Microsoft, Washington, United States) within 2 weeks of the CPETs and before the participants’ first three and last three consecutive CR classes (i.e., held on separate days)]. These classes were held at the same time each week for each participant (e.g., 10:00 h Tuesdays and Thursdays). Approximately 15 min of rest was provided before each submaximal exercise test, which was conducted by an exercise specialist (i.e., Registered Kinesiologist, Certified Exercise Physiologist, or Physiotherapist) in accordance with the American College of Sports Medicine (ACSM) guidelines (American College of Sports Medicine, 2017). During all submaximal exercise tests, heart rate and perceived exertion were measured by Polar HR monitors (FT1, Polar Electro, Kempele, Finland) and the Borg Rating of Perceived Exertion (RPE) scale (6–20 points). RPE values were calibrated for each participant before each test, by ensuring each participant began the tests at “no exertion at all” (point 6).

#### Modified Bruce Treadmill Test

Participants walked on a motorized treadmill (Precor C954i, Woodinville, Washington, United States) for 3-min stages of increasing speeds and grades (Stage 1: speed = 1.7 mph, grade = 0%; Stage 2: speed = 1.7 mph, grade = 5%; Stage 3: speed = 1.7 mph, grade = 10%; Stage 4: speed = 2.5 mph, grade = 12%; Stage 5: speed = 3.4 mph, grade = 14%; Stage 6: speed = 4.2 mph, grade = 15%; and Stage 7: speed = 5.0 mph, grade = 15%). Exercise HRs and RPEs were collected at the end of each 3-min stage. Tests were terminated: at the end of the stage during which participants reached 85% of their peak HR; when the participant requested to stop; or, the exercise specialist felt the participants could not safely continue. The speed and grade of the last completed stage were used to estimate V̇O_2_ from the ACSM walking equation (V̇O_2__peak_ = [speed (m/min) × 0.1] + [grade (decimal) × speed (m/min) × 1.8] + 3.5) (American College of Sports Medicine, 2017). The following equations were then used to extrapolate V̇O_2__peak_: (1) Slope (β) = (V̇O_2_ last stage – V̇O_2_ second last stage)/(HR last stage – HR second last stage); and, (2) V̇O_2__peak_ = V̇O_2_ last stage + β (HRpeak – HR last stage) (Heyward, 2010). Successful tests were defined as a participant completing at least two stages (to extrapolate V̇O_2__peak_) and reaching 85% of HRpeak upon completion of the final stage (Heyward, 2010; American College of Sports Medicine, 2017).

#### Astrand-Ryhming Cycle Ergometer Test

Participants cycled on an upright cycle ergometer (True Fitness, CS200, St. Louis, MO, United States at 50, 75, 100, 125, or 150 watts (or as close as possible to one of these wattages given a participant’s physical capabilities) while maintaining a pedal rate of 50 ± 5 rpm for the duration of the 6 min test. Exercise HR and RPE were collected at the end of minutes 5 and 6. The average of these HRs and the final watts were used to predict V̇O_2__peak_ from a nomogram (American College of Sports Medicine, 2017). An age correction factor was applied to this value (American College of Sports Medicine, 2017). Astrand et al. reported a correlation (*r*) of 0.78 between measured V̇O_2__peak_ from CPET and predicted V̇O_2__peak_ from the Astrand-Ryhming cycle ergometer test using the age-correction factor (Astrand, 1960). Successful tests were defined as a participant having completed the 6-min test at a workload to produce exercise HRs within the range of 120–170 bpm (the HR range required to predict V̇O_2__peak_ from the nomogram).

#### Chester Step Test

Using a step height (i.e., 15, 20, 25, or 30 cm) suitable to a participant’s functional level, participants stepped to a metronome beat of 15 steps per minute for 2 min following which HR and RPE were recorded. The step rate then increased to 20 steps per minute for 2 min following which HR and RPE were recorded. The test continued in this progressive manner to a maximum of five stages, until the end of the stage during which participants reached 85% of their peak HR (American College of Sports Medicine, 2017). The end of stage HRs, end of stage V̇O_2_ values from the Chester Step Test data sheet, and measured peak HRs from CPETs were entered into a spreadsheet program (Microsoft Excel, Microsoft Canada Inc., Mississauga, ON, Canada) to extrapolate V̇O_2__peak_ using the “trendline” function. Successful tests were defined as a participant having completed at least two stages (to extrapolate V̇O_2__peak_) and reaching 85% of HRpeak upon completion of the final stage.

#### Duke Activity Status Index

Participants completed the Duke Activity Status Index (DASI), a 12-item questionnaire that assesses daily activities such as personal care, ambulation, household tasks, sexual function and recreation activities at the beginning and following the 12-week CR program. Each activity is associated with an activity-specific value. Participants were asked to identify each activity they were able to perform. The values were summed and entered into the following equation to estimate peak exercise capacity_:_ V̇O_2__peak_ = (0.43 × DASI value) + 9.6 (Hlatky et al., 1989).

### Cardiovascular Rehabilitation

The CR program included: a CVD risk-factor modification consultation with a physiotherapist or registered nurse; medical assessment if deemed appropriate; referral to services which support CVD risk-factor management (i.e., vocational counseling, nutritional counseling, stress-management, social work and/or psychological support, if required); and, supervised exercise training sessions twice weekly in the Cardiac Prevention and Rehabilitation Center for 12 weeks. Risk-factor management addressed the importance of regular physical activity, healthy eating, smoking cessation, and stress management. Each exercise training session was 45–60 min in duration and included: (i) warm-up for 5–10 min; (ii) conditioning for 20–40 min at 40–85% HRR (RPE: 12–16); and, (iii) cool-down for 5–10 min (Canadian Association of Cardiac Rehabilitation, 2009). Resting (obtained following a 5 min rest period) and peak HRs obtained from the baseline CPETs were used to compute % heart rate reserve (HRR) (% HRR = [(HRpeak – HRrest × % intensity) + HRrest] (American College of Sports Medicine, 2017).

### Outcome Measures

#### Demographics and Medical Conditions

Demographics were retrieved from patients’ medical records and included age, sex, ethnicity, cardiovascular history and medication use.

#### Anthropometrics and Hemodynamics

Height was measured to the nearest 0.5 cm; body mass was measured to the nearest 0.1 kg at the beginning and completion of CR, and body mass index (BMI) was subsequently calculated (kg/m^2^). Waist circumference was measured to the nearest 0.5 cm at the midpoint between the lower costal margin and iliac crest while participants stood with arms at their sides, feet 25–30 cm apart and abdomen relaxed before and after completion of CR. Resting blood pressure and HR were measured using an automated, non-invasive blood pressure monitor (Bp-TRU, Canada; or, Welch Allyn, Canada) by CR professionals at baseline and following 12 weeks. All measures were performed in triplicate, and the averages reported.

#### Statistical Analysis

We used PASS to calculate the required sample size (Pass 13, NCSS, Utah, United States). Using a moderate correlation coefficient of 0.5, an alpha of 0.05 and adequate power (1-β) of 0.80, a sample size of 46 participants was required to assess the association of changes (from baseline to follow-up) in V̇O_2__peak_ following CR as predicted by three submaximal exercise tests and a self-reported activity status questionnaire with changes in V̇O_2__peak_ measured by CPET. We adjusted our sample size upward (*N* = 50) to account for a planned 10% loss to follow-up.

Analyses were performed using SPSS for Windows (version 24; IBM Corp., Armonk, NY, United States). All outcome variables were tested for normality using Shapiro–Wilk tests; baseline and follow-up V̇O_2__peak_ as predicted by the treadmill tests and DASI were not normally distributed. Descriptive analyses of the participants’ demographics and anthropometrics, cardiovascular conditions, medications and exercise test characteristics were performed. Paired *t*-tests and Wilcoxon signed–rank tests were used to examine changes in measured and predicted V̇O_2__peak_ for normally and non-normally distributed variables, respectively.

Pearson’s product-moment correlations were used to examine the association between measured and predicted V̇O_2__peak_ values obtained from CPET, submaximal exercise tests and the DASI. Linear regression analyses were used to examine whether proportional bias existed between measured and predicted V̇O_2__peak_ values. When there was no proportional bias (i.e., homoscedastic scatter), paired *t*-tests were used to examine whether systematic error existed between measured and predicted VO_2__peak_ values within participants at baseline and for changes. Bland-Altman plots were used for visual presentation of the agreement between CPET and submaximal exercise test and DASI V̇O_2__peak_ values (Bland and Altman, 1986; Ludbrook, 2010). If proportional bias was detected, a line of best fit was presented; this method is recommended when the mean and standard deviation of the differences are not constant (Bland and Altman, 1999; Ludbrook, 2010). Data are reported as mean ± standard deviation, and *p* < 0.05 was considered statistically significant.

## Results

### Participant Study Flow

A total of 74 participants were referred to the study coordinator. Fifty participants met the eligibility criteria and consented to participate; the remaining 24 candidates declined for the following reasons: lack of time (*n* = 6); illness or worsening of symptoms (*n* = 5); did not want to delay starting CR to complete baseline study measures (*n* = 4); lack of interest (*n* = 4); fearful of CPET (*n* = 2); no longer enrolled in CR (*n* = 1); not available for study appointments (*n* = 1); or, had pacemaker which limited exercise HRs (*n* = 1). Forty-six of the 50 participants completed the 12-week CR program and follow-up measures; this represents an 8% loss to follow-up.

### Participant Characteristics

Descriptive data for participants are shown in Table 1. The participants were predominately male, Caucasian and overweight, had poor to good exercise tolerance (American College of Sports Medicine, 2017) and multiple cardiovascular conditions. Most participants were taking anti-platelet, statin, β-blocker and ACE inhibitor medications. On average, participants exercised at between 72 and 82% of their HRR throughout the 12-week CR program.

**TABLE 1:** Participant characteristics.

	**Mean ± SD**
**Demographics and Anthropometrics (*n* = 47)**	
Age (years)	57 ± 11
Sex (% male)	77
Ethnicity (% white)	85
Height (cm)	171.0 ± 9.6
Body mass (kg)	83.8 ± 16.0
BMI (kg/m^2^)	28.7 ± 5.2
Waist circumference (cm)	100.2 ± 12.7
Resting systolic blood pressure (mmHg)	120 ± 14
Resting diastolic blood pressure (mmHg)	73 ± 9
Resting heart rate (bpm)	68 ± 12
**Cardiovascular conditions (*n* = 47)**	
Percutaneous coronary intervention, yes: no, *n*	30:17
STEMI, yes: no, *n*	15:32
Angiogram, yes: no, *n*	11:36
Non-STEMI, yes: no, *n*	8:39
Coronary artery bypass grafting surgery, yes: no, *n*	7:40
Angina, yes: no, *n*	6:41
Atrial fibrillation, yes: no, *n*	5:42
Previous myocardial infarction, yes: no, *n*	2:45
Mitral value surgery, yes: no, *n*	2:45
Aortic valve surgery, yes: no, *n*	1:46
Acute coronary syndrome, yes: no, *n*	1:46
Ablation, yes: no, *n*	1:46
**Medications (*n* = 49)**	
Anti-platelet, yes: no, *n*	48:1
Statins, yes: no, *n*	41:8
β-blocker, yes: no, *n*	33:16
ACE inhibitor, yes: no, *n*	20:29
Nitroglycerin, yes: no, *n*	18:31
Angiotensin II receptor antagonist, yes: no, *n*	3:46
Calcium channel blocker, yes: no, *n*	3:46
Thiazide diuretic, yes: no, *n*	3:46
PCSK9 inhibitor, yes: no, *n*	1:48

*ACE, angiotensin-converting enzyme; BMI, body mass index; ICD, implantable cardioverter-defibrillator; PCSK, proprotein convertase subtilisin/kexin; SD, standard deviation; STEMI, ST-elevation myocardial infarction.*

### Characteristics and Success of Baseline and Follow-Up CPET

At baseline, participants achieved an average peak HR and respiratory exchange ratio (RER) of 141.5 ± 22.9 bpm and 1.1 ± 0.1, respectively, during their CPET. At follow-up, participants achieved an average peak HR and RER of 146.2 ± 20.3 bpm and 1.1 ± 0.1, respectively, during their CPET. All CPETs were deemed successful, as these were peak tests which participants terminated when volitional fatigue was achieved. The number and percentage of successful treadmill, step and cycle tests as well as the number and percentage of participants who completed incremental treadmill and step test stages are shown in Table 2. Not all the same participants were successful or unsuccessful for different tests.

**TABLE 2:** The number and percentage of successful treadmill, step and cycle tests as well as participants who completed incremental treadmill and step test stages.

	**Baseline**	**Follow-up**
**Treadmill, *n* (%) successful**	34(69%)	31(67%)
**Stage of termination, *n* (%)**		
Stage 2	4(12%)	2(7%)
Stage 3	6(18%)	5(16%)
Stage 4	18(53%)	13(42%)
Stage 5	5(15%)	10(32%)
Stage 6	1(3%)	0(0%)
Stage 7	0(0%)	1(3%)
**Step, *n* (%) successful**	34(68%)	37(80%)
**Stage of termination, *n* (%)**		
Stage 2	4(12%)	3(8%)
Stage 3	15 (44)	10(27%)
Stage 4	13 (38)	18(49%)
Stage 5	2 (6)	6(16%)
**Cycle, *n* (%) successful**	19(38%)	21(46%)

*Stages 2 to 7 only are reported because successful tests were defined as the participant completing at least two stages.*

### Modified Bruce Treadmill Tests

#### Baseline

The baseline V̇O_2__peak_ values demonstrated by the submaximal treadmill tests are shown in Table 3. Significant positive correlations between the V̇O_2__peak_ values measured by CPET and those predicted by the treadmill tests were observed (*r* = 0.656, *p* < 0.001). A significant proportional bias and an apparent heteroscedastic limits of agreement between the V̇O_2__peak_ values measured by CPET and those predicted by the treadmill tests were observed (*F* = 11.029, *p* = 0.002, *n* = 34).

**TABLE 3:** Baseline, follow-up and change in V̇O_2__peak_, and analyses testing for proportional bias and associations between changes in V̇O_2__peak_ values measured by CPET and those predicted by estimation methods.

	**Baseline Mean ± SD *n***	**Follow-up Mean ± SD *n***	**Change Mean ± SD *n***	**Linear regression (to test for proportional bias in changes in V̇O_2__peak_ values measured by CPET and those predicted by estimation methods)**	**Correlations (to test for associations between changes in V̇O_2__peak_ values measured by CPET and those predicted by estimation methods)**
CPET (mL^.^kg^–1.^min^–1^)	25.9 ± 6.3	30.0 ± 6.7	3.6 ± 5.5^∗^	–	–
	*n* = 50	*n* = 46	*n* = 46		
Modified Bruce Treadmill Test (mL^.^kg^–1.^min^–1^)	29.3 ± 9.2	32.4 ± 13.9	3.5 ± 12.5	*F* = 27.382, *p* < 0.001	*r* = 0.202, *p* = 0.356
	*n* = 34	*n* = 31	*n* = 23		
Astrand-Ryhming Cycle Ergometer Test (mL^.^kg^–1^ min^–1^)	31.3 ± 7.0	32.5 ± 6.4	2.5 ± 5.7	*F* = 0.521, *p* = 0.487	*r* = −0.313, *p* = 0.322
	*n* = 19	*n* = 21	*n* = 12		
Chester Step Test (mL^.^kg^–1.^min^–1^)	30.4 ± 5.6	32.4 ± 6.3	1.9 ± 5.0	*F* = 0.188, *p* = 0.668	*r* = 0.127, *p* = 0.513
	*n* = 34	*n* = 37	*n* = 29		
Duke Activity Status Index (mL^.^kg^–1.^min^–1^)	29.5 ± 5.2	32.2 ± 4.1	2.3 ± 4.2^∗^	*F* = 2.562, *p* = 0.117	*r* = 0.085, *p* = 0.587
	*n* = 48	*n* = 45	*n* = 43		

*CPET, cardiopulmonary exercise test; SD, standard deviation. ^∗^, denotes significant change from baseline to follow-up.*

#### Follow-Up

**FIGURE 2:**
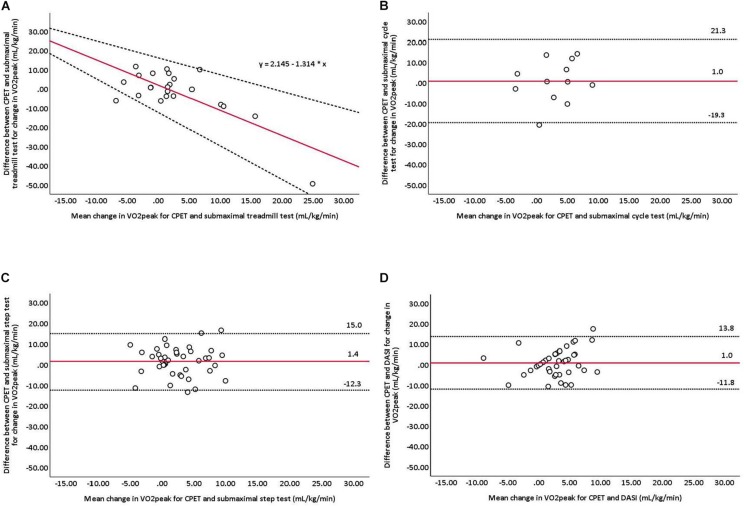
Bland-Altman plots comparing measured and predicted changes in V̇O_2peak_ for **(A)** Bruce treadmill, **(B)** Astrand-Ryhming cycle ergometer, and **(C)** Chester step tests, and **(D)** DASI. The red line represents mean differences between measured and predicted V̇O_2peak_. Dashed lines represent the limits of agreement (mean difference ±1.96 standard deviation). CPET, cardiopulmonary exercise testing; DASI, Duke Activity Status Index. A line of best fit is presented for the Bruce treadmill test as proportional bias was detected.

The follow-up and changes in V̇O_2__peak_ values demonstrated by the submaximal treadmill tests are shown in Table 3. No significant correlations between the change in V̇O_2__peak_ values measured by CPET and those predicted by the treadmill tests were observed (*r* = 0.202, *p* = 0.356). A significant proportional bias between the change in V̇O_2__peak_ values measured by CPET and those predicted by the treadmill tests was observed (*F* = 27.382, *p* < 0.001, *n* = 23). Proportional bias remained with the removal of an outlier (i.e., a −49 mL^.^kg^–1.^min^–1^ difference in the change in V̇O_2__peak_ measured by CPET and that predicted by the treadmill test). Bland-Altman plots showing the level of agreement between measured and predicted changes in the V̇O_2__peak_ values are shown in Figure 2. A total of 22% of differences in changes in V̇O_2__peak_ values fell within ± 1.75 mL^.^kg^–1.^min^–1^ (0.5 METs) of the mean bias for the treadmill tests.

### Astrand-Ryhming Cycle Ergometer Tests

#### Baseline

The baseline V̇O_2__peak_ values demonstrated by the submaximal cycle tests are shown in Table 3. No significant correlations between the V̇O_2__peak_ values measured by CPET and those predicted by the cycle tests were observed (*r* = −0.003, *p* = 0.991). No significant differences between the V̇O_2__peak_ values predicted by the cycle tests and those measured by CPET were observed (31.3 ± 7.0 vs. 27.5 ± 5.3 mL^.^kg^–1.^min^–1^, *p* = 0.077, *n* = 19); the mean bias and 95% limits of agreement was 3.8 mL^.^kg^–1.^min^–1^ (21.0, −13.4).

#### Follow-Up

The follow-up and changes in V̇O_2__peak_ values demonstrated by the submaximal cycle tests are shown in Table 3. No significant correlations between the change in V̇O_2__peak_ values measured by CPET and those predicted by the cycle tests were observed (*r* = −0.313, *p* = 0.322). No significant differences in the change in V̇O_2__peak_ values measured by CPET and those predicted by the cycle tests were observed (Δ3.5 ± 7.1 vs. Δ2.5 ± 5.7 mL^.^kg^–1.^min^–1^, *p* = 0.751; *n* = 12). Bland-Altman plots showing the level of agreement between measured and predicted changes in the V̇O_2__peak_ values are shown in Figure 2. A total of 50% of differences in changes in V̇O_2__peak_ values fell within ± 1.75 mL^.^kg^–1.^min^–1^ (0.5 METs) of the mean bias for the cycle tests.

### Chester Step Tests

#### Baseline

The baseline V̇O_2__peak_ values demonstrated by the submaximal step tests are shown in Table 3. Significant positive correlations between the V̇O_2__peak_ values measured by CPET and those predicted by the step tests were observed (*r* = 0.693, *p* < 0.001). The step tests significantly overestimated V̇O_2__peak_ values when compared to CPET (30.4 ± 5.6 vs. 26.3 ± 5.6 mL^.^kg^–1.^min^–1^, *p* < 0.001, *n* = 34); the mean bias and 95% limits of agreement was 4.1 mL^.^kg^–1.^min^–1^ (12.7, −4.5).

#### Follow-Up

The follow-up and changes in V̇O_2__peak_ values demonstrated by the submaximal step tests are shown in Table 3. No significant correlations between the change in V̇O_2__peak_ values measured by CPET and those predicted by the step tests were observed (*r* = 0.127, *p* = 0.513). No significant differences in the change in V̇O_2__peak_ values measured by CPET and those predicted by the step tests were observed (Δ3.3 ± 5.5 vs. Δ1.9 ± 5.0 mL^.^kg^–1.^min^–1^, *p* = 0.300; *n* = 29). Bland-Altman plots showing the level of agreement between measured and predicted changes in the V̇O_2__peak_ values are shown in Figure 2. A total of 38% of differences in changes in V̇O_2__peak_ values fell within ± 1.75 mL^.^kg^–1.^min^–1^ (0.5 METs) of the mean bias for the step tests.

### Duke Activity Status Index

#### Baseline

The baseline V̇O_2__peak_ values demonstrated by the DASI are shown in Table 3. Significant positive correlations between the V̇O_2__peak_ values measured by CPET and those predicted by the DASI were observed (*r* = 0.379, *p* = 0.008). The DASI significantly overestimated V̇O_2__peak_ values when compared to CPET (29.5 ± 5.2 vs. 25.8 ± 6.4 mL^.^kg^–1.^min^–1^, *p* < 0.001, *n* = 48); the mean bias and 95% limits of agreement was 3.6 mL^.^kg^–1.^min^–1^ (16.4, −9.2).

#### Follow-Up

The follow-up and changes in V̇O_2__peak_ values demonstrated by the DASI are shown in Table 3. No significant correlations between the change in V̇O_2__peak_ values measured by CPET and those predicted by the DASI were observed (*r* = 0.085, *p* = 0.587). No significant differences in the change in V̇O_2__peak_ values measured by CPET and those predicted by the DASI were observed (Δ3.3 ± 5.4 vs. Δ2.3 ± 4.2 mL^.^kg^–1.^min^–1^, *p* = 0.307; *n* = 43). Bland-Altman plots showing the level of agreement between measured and predicted changes in the V̇O_2__peak_ values are shown in Figure 2. A total of 58% of differences in changes in V̇O_2__peak_ values fell within ± 1.75 mL^.^kg^–1.^min^–1^ (0.5 METs) of the mean bias for the DASI.

### Adverse Events

One participant was withdrawn from the study due to hemoptysis (unidentified source) during the CR phase. This adverse event was deemed serious, unexpected and unrelated to the study. No adverse events occurred during the baseline or follow-up CPET; reasons for test termination were expected and included: fatigue, shortness of breath, chest pain (possible angina), leg pain, dizziness, light-headedness, and lack of motivation. No severe adverse events occurred during the baseline or follow-up submaximal exercise tests. The mild and moderate symptoms experienced during these exercise tests are summarized in Table 4.

**TABLE 4:** Symptoms (moderate and mild) experienced during submaximal exercise tests.

	**Baseline**			**Follow-up**		
	**Treadmill**	**Cycle ergometer**	**Step**	**Treadmill**	**Cycle ergometer**	**Step**
**Moderate**						
Tightness in chest	1	–	–	–	–	–
**Mild**						
Leg tightness/pain (hip, groin, thighs, quads, knees, calves)	8	4	6	6	3	3
Dizziness	1	1	1	–	–	1
Lightheaded	–	–	–	1	–	–
Burning in chest	–	–	1	–	–	–
Shortness of breath	–	–	1	2	–	–
Foot cramping	–	–	1	–	–	1
Heart palpitations	–	–	1	–	–	–
Heart burn	–	–	1	–	–	–
Soreness at vein graft site	–	–	1	–	–	–
Edema in calves	–	–	–	1	–	–
Sciatic pain	–	–	–	1	–	–
TOTAL *n* (%)	10 (20)	5 (10)	13 (27)	11 (24)	3 (7)	5 (11)

## Discussion

This study is the first, to our knowledge, to assess changes in V̇O_2__peak_ following CR as predicted by three submaximal exercise tests (i.e., treadmill, cycle and step) and a self-reported activity status questionnaire (i.e., DASI) in comparison with gold-standard CPET. In this pre-post experimental study, we observed that most patients successfully completed the step tests (baseline: 68%; follow-up: 80%), while few patients successfully completed the cycle tests (baseline: 38%; follow-up: 46%). Significant improvements from baseline to follow-up in V̇O_2__peak_ were observed with CPET (3.6 mL^.^kg^–1.^min^–1^) and predicted by the DASI (2.3 mL^.^kg^–1.^min^–1^), yet none of the submaximal exercise tests. Bland-Altman plots revealed significant proportional bias between the change in V̇O_2__peak_ values measured by CPET and those predicted by the treadmill test. No significant differences between the change in V̇O_2__peak_ values measured by CPET and those predicted by the cycle and step tests or DASI were observed; the mean bias and limits of agreement were 1.0 mL^.^kg^–1.^min^–1^ (21.3, −19.3), 1.4 mL^.^kg^–1.^min^–1^ (15.0, −12.3) and 1.0 mL^.^kg^–1.^min^–1^ (13.8, −11.8), respectively. Given the greater number of successful tests, no serious adverse events and acceptable group-level mean bias, the Chester step test appears to be a safe and valid method for assessing mean, not individual-level changes in V̇O_2__peak_ among patients with CVD in a CR program when compared to gold-standard CPET. The DASI also appears to be a practical, cost-effective and valid method for assessing mean, not individual-level changes in V̇O_2__peak_ among patients with CVD in a CR program.

### Modified Bruce Treadmill Tests

We used the well-known ACSM walking equation to predict V̇O_2__peak_ at baseline and follow-up using the final speeds and grades obtained from the treadmill tests (American College of Sports Medicine, 2017). Proportional bias was observed between the change in V̇O_2__peak_ values measured by CPET and those predicted by the treadmill test, such that there was greater disagreement between the measured and predicted tests with greater V̇O_2__peak_ values. Our data, therefore, indicate that the treadmill test is not a valid tool for assessing changes in V̇O_2__peak_ in patients with CVD completing a CR program. Potential sources of error contributing to the proportional bias may have included: the curvilinear relationship between oxygen consumption, HR and workload at near-maximal effort (the treadmill test assumes a linear relationship between HR and workload); (Looney et al., 2019) and, the placing of their hands on the treadmill rails for balance by several participants, during their last or second last stage. The purpose of this study was to assess *a priori* changes in V̇O_2__peak_ following CR as predicted by the submaximal modified Bruce treadmill test (using the ACSM walking equation) when compared to CPET. Future studies could explore the validity of all available equations developed for submaximal treadmill tests in those with CVD in predicting changes in V̇O_2__peak_ following CR against gold-standard CPET.

### Astrand-Ryhming Cycle Ergometer Tests

Very few participants successfully completed the cycle tests as they were not able to continuously cycle for 6 min at a sufficient workload (likely due to poor muscle strength and endurance) to produce exercise HRs within the range of 120–170 bpm (the HR range required to predict V̇O_2__peak_ from the Astrand-Ryhming nomogram). More participants (46 vs. 38%) successfully completed the follow-up than baseline cycle tests; these improvements may reflect the use of CR exercise modalities which included cycling. As most (67%) participants were taking β-blockers, it is likely that these HR blunting medications prevented participants from achieving exercise HRs on the nomogram. There is a need to develop a valid nomogram or prediction equation to evaluate changes in V̇O_2__peak_ values using the Astrand-Ryhming cycle test in patients with CVD who frequently take HR blunting medications; this is beyond the scope of this validation study and would require a larger sample size.

### Chester Step Tests

The mean bias in changes in V̇O_2__peak_ was 1.4 mL^.^kg^–1.^min^–1^ (i.e., 0.4 METs) for the step test; there were no significant differences between CPET and the step test. A modest increase of 1.75 to 3.5 mL^.^kg^–1.^min^–1^ (i.e., 0.5 to 1 METs) following CR is considered clinically important (Grace et al., 2016), particularly because an increase of this magnitude is strongly associated with lower morbidity and mortality (Myers et al., 2002). The step test, thus, appears to be appropriate for estimating mean changes in V̇O_2__peak_ following CR as the observed mean bias was less than 1.75 mL^.^kg^–1.^min^–1^. However, caution is warranted in using the step test to estimate changes in V̇O_2__peak_ following CR on an individual-level given only 38% of the changes in V̇O_2__peak_ values fell within ± 1.75 mL^.^kg^–1.^min^–1^ (0.5 METs) of the mean bias for the step test, and we observed wide limits of agreement (15, −12 mL^.^kg^–1.^min^–1^). Our findings are consistent with classic studies showing that individual predictions of V̇O_2__peak_ using progressive stepping tests are liable to considerable error (Fitchett, 1985). Potential sources of error contributing to the wide limits of agreement may have included: the curvilinear relationship between oxygen consumption, HR and workload at near-maximal effort (the step test assumes a linear relationship between HR and workload); and, the participants’ inability to maintain the correct stepping tempo and technique, affecting mechanical efficiency (Bennett et al., 2016). From our experience, participants found this exercise test modality the most challenging, particularly for those fearful of falling or who experienced difficulties with foot coordination. To reduce error, we used: a statistical line of best fit to remove the potential variability in drawing a visual line of best fit; measured peak HR from CPET (Buckley et al., 2004); and, Polar HR monitoring instead of manual HR recordings.

### Duke Activity Status Index

The mean bias in changes in V̇O_2__peak_ was 1.0 mL^.^kg^–1.^min^–1^ (i.e., 0.3 METs) for the DASI; there were no significant differences between CPET and the DASI. As mentioned above, a modest increase of 1.75 to 3.5 mL^.^kg^–1.^min^–1^ following CR is considered clinically important (Grace et al., 2016). The DASI, thus, appears to be appropriate for estimating mean changes in V̇O_2__peak_ following CR as the observed mean bias was less than 1.75 mL^.^kg^–1.^min^–1^. However, one must exercise caution when using the DASI to estimate individual-level changes in V̇O_2__peak_ following CR given the wide limits of agreement (13.8, −11.8 mL^.^kg^–1.^min^–1^), and only 58% of the changes in V̇O_2__peak_ values fell within ± 1.75 mL^.^kg^–1.^min^–1^ (0.5 METs) of the mean bias for the DASI. Potential sources of error contributing to the wide limits of agreement may have included: our measured V̇O_2__peak_ values were obtained from CPET on a treadmill, while the DASI was validated against CPET on a cycle ergometer (Hlatky et al., 1989); V̇O_2__peak_ values obtained from treadmill exercise may be up to 10% higher (Hermansen and Saltin, 1969). Further, several participants found the DASI questions challenging to answer, particularly those who had not tried an activity due to lack of interest (e.g., women who were not interested in vigorous sports such as singles tennis), or other scenarios (e.g., seasonal activities not performed during the time of year participants completed the questionnaire, activities participants had been advised not to engage in by their physician).

### Safety

We meticulously tracked all mild, moderate and severe symptoms and adverse events throughout this study. The greatest and fewest number of symptoms (i.e., tightness in chest, leg tightness/pain, dizziness, lightheaded, foot cramping, heart palpitations and burning, edema, sciatic pain) occurred during the treadmill (20–24%) and cycle (7–10%) testing, respectively. Given the mild to moderate nature of these symptoms, which are expected with exercise testing in patients with CVD, our findings suggest that submaximal exercise testing does not pose any greater risk than peak CPET. The risk of peak exercise testing is low, with approximately 6 cardiovascular events per 10,000 tests (0.06%) occurring at >85% peak HR (American College of Sports Medicine, 2017). In other studies examining the use of submaximal exercise tests in predicting V̇O_2__peak_ when compared to CPET at a single time point in patients with CVD, potential adverse events were neither reported nor discussed (Milani et al., 1996, 1998; Hautala et al., 2013; Papini et al., 2017).

### Strengths and Limitations

Our study has several strengths. It is the first study to assess changes in V̇O_2__peak_ following CR as predicted by submaximal exercise tests of varying modalities and a self-reported activity status questionnaire in comparison with gold-standard CPET. This is particularly important as CPET is often impractical due to the costs, time, expertise and technological resources required. Consequently, CPET is underutilized in many CR settings. Second, the submaximal exercise tests were performed in a random order before the participants’ first three and last three consecutive CR classes to reduce order bias. Third, we meticulously tracked medications to ensure similar medications were taken before CPET and submaximal exercise testing to account for the HR blunting effects of β-blockers (Reed and Pipe, 2016). Finally, all peak and submaximal tests were performed on the same treadmill, cycle or step. We used actual peak HR from the CPET for submaximal test termination criteria (i.e., 85% HRpeak for the treadmill and step tests) as age-predicted HRpeak have been shown to overestimate actual HRpeak and there’s no gold-standard prediction equation to use; further, we used actual peak HR from the CPET to first evaluate these submaximal tests under “best case” conditions (i.e., when both CPET and submaximal exercise tests are available) (Buckley et al., 2004).

Several limitations should be mentioned. First, the generalizability of our findings to females with CVD participating in CR is limited as 77% of our sample was male – typical of the under-representation of females enrolling in and adhering to CR (Samayoa et al., 2014; Oosenbrug et al., 2016). Future studies examining sex differences in V̇O_2__peak_ values predicted by submaximal exercise tests of varying modalities in comparison to CPET are needed as previous studies have reported different mean bias values for apparently healthy females when compared to males for the Chester step test (Sykes and Roberts, 2004). Second, few participants successfully completed the cycle tests (baseline: 38%; follow-up: 46%) which limited our power to predict changes in V̇O_2__peak_ when compared to CPET. The motivation required to complete a 6-min single-stage cycle test may have been limiting when compared to 2–3 min multi-stage treadmill and step tests, thus negatively impacting the success rates of the cycle tests. Third, successful step tests were defined as a participant having completed at least two stages (to extrapolate V̇O_2__peak_ using the line of best fit function) and reaching 85% of HRpeak upon completion of the final stage. It is reasonable to assume that including a minimum of two compared to three stages for a line of best fit calculation may contribute to increased variance. However, this was not observed in the current study (data not presented) likely due to the few number of participants (*n* = 3) who would have been removed from such analyses. Fourth, all participants were recruited from a single tertiary-care cardiovascular health center; future research would benefit from a multi-center trial evaluating changes in V̇O_2__peak_ following CR as predicted by submaximal exercise tests of varying modalities and the DASI when compared to CPET. Finally and importantly, this study was not powered to determine safety of submaximal exercise testing

in CR settings; future, larger, multi-center trials are needed for such an evaluation.

## Conclusion

The challenge in most rehabilitation settings is to induce and maintain subtle but sustained changes in regular patterns of physical activity – within a population that has typically been inactive. Thus, there is a need for simple, inexpensive approaches to both the evaluation of exercise capacity and the provision of guidance regarding physical activity in rehabilitation programs (Reed and Pipe, 2016). In situations where CPET is not safe, practical or feasible (as is the case for many CR programs), we conclude that the Chester step tests and DASI appear to be a valid and safe submaximal tools for predicting mean, not individual-level changes in V̇O_2__peak_ following CR. Such testing may be appropriate when only an estimate of V̇O_2__peak_ is required, as is typical for a substantial number of CR settings, but not when an exact measure is needed.

## Data Availability Statement

The datasets generated for this study are available on request to the corresponding author.

## Ethics Statement

The studies involving human participants were reviewed and approved by the Ottawa Health Science Network Research Ethics Board (Protocol #: 20150443-01H). The patients/participants provided their written informed consent to participate in this study.

## Author Contributions

JR, JH, BM, and AP conceived and designed the study. JR, LC, CC, and JH conducted the submaximal exercise tests. BM reviewed and interpreted the ECG and medication data. JR, LC, TT, and JB analyzed and interpreted the data. JR led the writing of the manuscript and had the primary responsibility for the final manuscript. All authors edited and critically reviewed the manuscript, read, and approved the final manuscript.

## Disclaimer

The results of the present study do not constitute endorsement by the ACSM. The results of the study are presented clearly, honestly, and without fabrication, falsification, or inappropriate data manipulation.

## Conflict of Interest

The authors declare that the research was conducted in the absence of any commercial or financial relationships that could be construed as a potential conflict of interest.
